# Dietary l-Arginine Supplementation Protects Weanling Pigs from Deoxynivalenol-Induced Toxicity

**DOI:** 10.3390/toxins7041341

**Published:** 2015-04-15

**Authors:** Li Wu, Peng Liao, Liuqin He, Zemeng Feng, Wenkai Ren, Jie Yin, Jielin Duan, Tiejun Li, Yulong Yin

**Affiliations:** Key Laboratory of Agro-ecological Processes in Subtropical Region, Institute of Subtropical Agriculture, Chinese Academy of Sciences, Changsha 410125, Hunan, China; E-Mails: adonis5@163.com (L.W.); hlq285687180@126.com (L.H.); zemengfeng2006@163.com (Z.F.); renwenkai19@126.com (W.R.); yinjie2014@126.com (J.Y.); djielin119@163.com (J.D.)

**Keywords:** deoxynivalenol (DON), l-arginine, amino acid transporter, weanling pigs

## Abstract

This study was conducted to determine the positive effects of dietary supplementation with l-arginine (Arg) on piglets fed a deoxynivalenol (DON)-contaminated diet. A total of eighteen, 28-day-old healthy weanling pigs were randomly assigned into one of three groups: uncontaminated basal diet (control group), 6 mg/kg DON-contaminated diet (DON group) and 6 mg/kg DON + 1% l-arginine (DON + ARG group). After 21 days of Arg supplementation, piglets in the DON and DON + ARG groups were challenged by feeding 6 mg/kg DON-contaminated diet for seven days. The results showed that DON resulted in damage to piglets. However, clinical parameters, including jejunal morphology, amino acid concentrations in the serum, jejunum and ileum, were improved by Arg (*p* < 0.05). Furthermore, the mRNA levels for sodium-glucose transporter-1 (SGLT-1), glucose transporter type-2 (GLUT-2) and y^+^l-type amino acid transporter-1 (y^+^LAT-1) were downregulated in the DON group, but the values were increased in the DON + ARG group (*p* < 0.05). Collectively, these results indicate that dietary supplementation with Arg exerts a protective role in pigs fed DON-contaminated diets.

## 1. Introduction

*Fusarium* infection of wheat, barley and corn with concurrent production of deoxynivalenol (DON) and other trichothecene mycotoxins is an increasingly important food safety issue worldwide [1,2]. Many published papers show the toxic effects of DON on animals, mainly impairing the immune system and the health status of the gastrointestinal tract and the brain [1,2,3,4,5,6,7]. Ingestion of DON has also been associated with gastroenteritis, as reflected by nausea, emesis, diarrhea, anorexia and gastrointestinal hemorrhaging [4]. Considering that the gastrointestinal tract and the immune system of pigs are not vastly different from those of humans, the pig can be regarded as a good model that can be applied to humans [3,4]. Intestine is the primary target of DON. As a dynamic barrier, the intestine is responsible primarily for digestion and absorption of dietary nutrients, as well as excluding potential pathogens and toxins [5]. The absorption of DON occurs in the proximal part of jejunum in which DON produces its subsequent toxicology [6,7], and then, DON widely spreads to various tissues. Therefore, the adverse effects of DON on intestine involve pathological changes, including abnormal permeability and reduced expression of nutrient transporters.

l-arginine (Arg) promotes intestinal growth, development and maturation [8,9,10]. It has been reported that Arg stimulates phagocyte activity and accelerates endotoxic elimination in the gut [11]. Arg also benefits mucosal microcirculation and absorption [12,13,14]. To date, no study has been conducted to determine the roles of Arg in DON contamination. According to the understanding of DON toxicology and the biological function of Arg, we hypothesized that dietary Arg may alleviate the functional impairment of intestine brought about by DON in weanling piglets.

## 2. Results

### 2.1. Analysis of Moldy Corn

Table 1 summarizes the mycotoxin content in contaminated and non-contaminated feed mixture. The resulting ground moldy corn was determined to contain 0.52 mg/kg DON. Before the challenge, the moldy corn was added to the diet, providing DON at 6 mg/kg.

### 2.2. Free Amino Acid Concentration in Serum

Table 2 summarizes the concentration of free amino acids in serum after challenge. The trends in Arg, histidine, methionine and threonine were similar. The values in the DON group were the lowest among three groups, while only slight differences were observed between the DON + ARG and DON groups (*p* > 0.05). After DON exposure, leucine concentration was reduced markedly (*p* < 0.05), but there was no significant difference between the DON and DON + ARG groups (*p* > 0.05). Similar results were obtained for valine, lysine, tryptophan and ornithine. Serum γ-amino-*n*-butyric acid concentrations in the DON group were significantly higher than those in the other groups. Compared to the other two groups, there was a significant decline after DON challenge with regard to isoleucine value (*p* < 0.01). However, marked differences were not found in the rest of the other free amino acids in the serum.

**Table 1:** Mycotoxin content in contaminated and non-contaminated feed mixture.

Catalogue	AFB1 ^1^ (ppb)	ZEN ^2^ (ppm)	OCH ^3^ (ppb)	FB1 ^4^ (ppm)	T-2 (ppm)	DON ^5^ (ppm)
Limit of detection	0.05	0.01	0.5	0.05	0.1	0.1
Basal feed	undetected	0.863	3.74	0.65	undetected	0.52
Contaminated feed	undetected	0.697	4.63	0.74	undetected	-

^1^ Aflatoxin B1; ^2^ zearalenone; ^3^ ochratoxins; ^4^ fumonisins; ^5^ deoxynivalenol. The contents of mycotoxins in the diet were detected by liquid chromatography (Beijing Taileqi, Beijing, China).

**Table 2:** Concentrations of free amino acid in the pig serum after deoxynivalenol (DON) challenge (*n* = 6).

Items	Control ^1^	DON ^2^	DON + ARG ^3^	SEM	*p*-Value
Arginine	60.77 ± 11.86 ^b^	41.79 ± 6.76 ^a^	50.72 ± 6.76 ^ab^	2.707	0.007
Histidine	40.27 ± 10.86 ^b^	25.66 ± 7.64 ^a^	30.70 ± 4.31 ^ab^	2.312	0.021
Isoleucine	28.21 ± 6.59 ^b^	12.04 ± 6.36 ^a^	21.21 ± 4.58^b^	2.072	0.001
Leucine	49.18 ± 12.62 ^b^	31.83 ± 3.14 ^a^	36.84 ± 8.20 ^a^	2.644	0.012
Lysine	85.28 ± 9.07 ^b^	66.07 ± 8.10 ^a^	69.10 ± 13.32 ^a^	3.083	0.013
Methionine	32.93 ± 5.84 ^b^	21.38 ± 8.82 ^a^	26.77 ± 3.66 ^ab^	1.832	0.025
Phenylalanine	28.29 ± 7.55	21.53 ± 3.69	23.90 ± 3.47	1.347	0.110
Threonine	55.61 ± 17.86 ^b^	34.44 ± 8.56 ^a^	34.24 ± 16.89 ^ab^	4.121	0.040
Tryptophan	38.17 ± 7.46 ^b^	22.19 ± 4.83 ^a^	25.30 ± 5.14 ^a^	2.130	0.001
Valine	55.05 ± 20.95 ^b^	24.95 ± 7.36 ^a^	34.79 ± 10.88 ^a^	4.386	0.007
γ-amino-*n*-butyric acid	0.08 ± 0.03 ^b^	0.11 ± 0.02 ^a^	0.10 ± 0.03 ^b^	0.007	0.108
Glycine	156.07 ± 29.8	130.56 ± 20.5	136.14 ± 43.2	7.679	0.385
Serine	34.94 ± 6.40	34.23 ± 14.11	33.07 ± 15.92	2.846	0.968
Taurine	68.83 ± 12.01	50.72 ± 14.28	59.56 ± 15.74	3.599	0.118
Tyrosine	32.82 ± 10.44	20.09 ± 5.74	26.91 ± 9.76	2.338	0.076
Asparagine	21.72 ± 5.55	22.79 ± 12.02	23.79 ± 10.57	2.175	0.935
Aspartic acid	5.80 ± 1.11	4.37 ± 0.98	6.33 ± 2.10	0.385	0.093
Citrulline	18.25 ± 5.65	12.69 ± 4.34	15.48 ± 2.34	1.106	0.119
Glutamic acid	48.45 ± 9.06	49.84 ± 4.69	44.39 ± 8.98	1.825	0.476
Glutamine	326.46 ± 52.7	255.06 ± 50.0	309.51 ± 55.5	13.829	0.080
Ornithine	23.36 ± 5.44 ^b^	14.66 ± 3.70 ^a^	17.30 ± 4.48 ^a^	1.347	0.015
Cystine	1.02 ± 0.54	0.50 ± 0.29	0.63 ± 0.43	0.110	0.134
α-amino-*n*-butyric acid	18.75 ± 7.81	16.30 ± 3.37	13.45 ± 6.16	1.442	0.343
Alanine	131.44 ± 36.2	136.38 ± 39.3	138.11 ± 36.0	8.264	0.949
hydroxy-l-proline	23.37 ± 7.88	21.28 ± 9.00	19.41 ± 11.35	2.144	0.775
1-methyl-l-histidine	5.19 ± 1.29 ^b^	3.94 ± 2.06 ^a^	4.17 ± 0.32 ^a^	0.340	0.298
3-methyl-l-histidine	0.86 ± 0.13	0.73 ± 0.37	0.84 ± 0.15	0.055	0.640
Proline	49.11 ± 12.39	53.65 ± 12.41	39.29 ± 10.37	2.983	0.131

^a,b^ Means in the same row with different superscripts differ (*p* < 0.05). ^1^ Control = basal diet; ^2^ DON = basal diet + 6 mg/kg deoxynivalenol; and ^3^ DON + ARG = basal diet + 6 mg/kg deoxynivalenol + 1% l-arginine. Results are expressed as the means ± SEM for six animals.

### 2.3. Free AA Concentrations in Ileum and Jejunum

Table 3 and Table 4 show free amino acid (AA) concentrations in ileum and jejunum after challenge, respectively. In the ileum, there was a significant change in isoleucine concentration among the three groups (*p* < 0.01); compared to other groups, the value in the DON group was the lowest. After DON challenge, the concentration of some AA decreased considerably, including Arg, histidine, leucine, methionine, phenylalanine, threonine, valine, serine, taurine, tyrosine, citrulline and ornithine. Meanwhile, the declines in free AA concentrations in the DON + ARG group were not notable. Similarly, in the jejunum, the concentrations of Arg, isoleucine, leucine, lysine, methionine, threonine, tryptophan, valine, taurine, tyrosine, asparagine, glutamine and ornithine were reduced significantly in the DON group. Only a slight increase was found in the DON + ARG group, compared to the DON group. There is no significant difference between the DON + ARG and control groups in the concentrations of serine, glutamic acid, alanine or 3-methyl-l-histidine.

**Table 3:** Concentrations of free amino acids in the ileum after deoxynivalenol (DON) challenge (*n* = 6).

Items	Control ^1^	DON ^2^	DON + ARG ^3^	SEM	*p*-Value
Arginine	18.30 ± 2.47 ^b^	13.59 ± 2.46 ^a^	14.67 ± 2.38 ^a^	0.728	0.011
Histidine	6.44 ± 0.48 ^b^	5.12 ± 0.74 ^a^	5.23 ± 0.78 ^a^	0.208	0.007
Isoleucine	6.67 ± 0.30 ^c^	4.42 ± 0.74 ^a^	5.20 ± 0.66 ^b^	0.262	0.000
Leucine	15.61 ± 0.84 ^b^	11.64 ± 1.36 ^a^	12.80 ± 2.23 ^a^	0.535	0.002
Lysine	27.47 ± 4.34	23.14 ± 6.05	23.39 ± 2.79	1.125	0.220
Methionine	9.35 ± 0.61 ^b^	6.68 ± 0.97 ^a^	7.52 ± 1.67 ^a^	0.374	0.004
Phenylalanine	8.68 ± 0.59 ^b^	6.83 ± 1.32 ^a^	7.11 ± 1.29 ^a^	0.318	0.025
Threonine	14.31 ± 1.20 ^b^	10.98 ± 1.56 ^a^	12.08 ± 1.89 ^a^	0.484	0.007
Tryptophan	1.50 ± 0.31	1.13 ± 0.25	1.34 ± 0.29	0.073	0.104
Valine	12.51 ± 0.92 ^b^	8.17 ± 1.17 ^a^	8.96 ± 1.01 ^a^	0.512	0.000
γ-amino-*n*-butyric acid	0.01 ± 0.00	0.02 ± 0.01	0.01 ± 0.00	0.002	0.327
Glycine	53.28 ± 4.12	50.37 ± 11.81	48.60 ± 6.09	1.840	0.605
Serine	24.59 ± 2.33 ^b^	19.66 ± 2.51 ^a^	19.76 ± 2.17 ^a^	0.762	0.003
Taurine	45.72 ± 3.62 ^b^	35.96 ± 5.71 ^a^	34.28 ± 3.20 ^a^	1.552	0.001
Tyrosine	9.44 ± 0.56 ^b^	6.64 ± 0.93 ^a^	6.99 ± 1.43 ^a^	0.379	0.001
Asparagine	12.31 ± 1.17	10.28 ± 2.41	11.69 ± 2.37	0.502	0.250
Aspartic acid	9.57 ± 2.17	8.91 ± 2.01	10.33 ± 1.70	0.458	0.477
Citrulline	3.69 ± 0.95 ^b^	2.04 ± 0.51 ^a^	2.31 ± 0.42 ^a^	0.229	0.001
Glutamic acid	42.43 ± 8.22	45.03 ± 9.81	45.66 ± 11.06	2.189	0.833
Glutamine	271.15 ± 22.2	232.67 ± 57.6	227.00 ± 32.6	10.109	0.153
Ornithine	4.58 ± 0.47 ^b^	2.79 ± 0.52 ^a^	2.86 ± 0.41 ^a^	0.226	0.000
Cystine	0.66 ± 0.16	0.76 ± 0.18	0.55 ± 0.23	0.047	0.205
α-amino-*n*-butyric acid	281.41 ± 87.9	211.73 ± 24.5	275.10 ± 59.1	15.863	0.139
Alanine	32.10 ± 4.37	25.92 ± 7.66	30.41 ± 6.15	1.514	0.237
hydroxy-l-proline	13.78 ± 2.24	13.00 ± 2.97	12.13 ± 1.67	0.547	0.498
1-methyl-l-histidine	0.10 ± 0.05	0.07 ± 0.04	0.08 ± 0.04	0.010	0.540
3-methyl-l-histidine	0.03 ± 0.01	0.02 ± 0.01	0.02 ± 0.01	0.004	0.493
Proline	26.44 ± 2.30	23.83 ± 2.69	24.36 ± 2.40	0.612	0.188

^a–c^ Means in the same row with different superscripts differ (*p* < 0.05). ^1^ Control = basal diet; ^2^ DON = basal diet + 6 mg/kg deoxynivalenol; and ^3^ DON + ARG = basal diet + 6 mg/kg deoxynivalenol + 1% l-arginine. Results are expressed as the means ± SEM for six animals.

**Table 4:** Concentrations of free amino acids in the jejunum after deoxynivalenol (DON) challenge (*n* = 6).

Items	Control ^1^	DON ^2^	DON + ARG ^3^	SEM	*p*-Value
Arginine	17.08 ± 1.85 ^a^	11.46 ± 2.86 ^b^	12.62 ± 1.84 ^b^	0.769	0.001
Histidine	5.20 ± 2.63	3.91 ± 1.04	4.39 ± 0.64	0.392	0.424
Isoleucine	6.26 ± 1.01 ^a^	4.22 ± 1.06 ^b^	4.26 ± 0.70 ^b^	0.310	0.002
Leucine	16.56 ± 2.09 ^a^	11.55 ± 2.96 ^b^	13.00 ± 2.47 ^b^	0.758	0.011
Lysine	28.76 ± 5.27 ^a^	20.32 ± 3.96 ^b^	20.62 ± 2.19 ^b^	1.299	0.003
Methionine	11.74 ± 1.63 ^a^	6.28 ± 1.93 ^b^	6.77 ± 1.64 ^b^	0.711	0.000
Phenylalanine	8.70 ± 4.41	6.95 ± 1.87	7.43 ± 1.47	0.489	0.571
Threonine	13.53 ± 1.17 ^a^	9.40 ± 2.01 ^b^	10.73 ± 2.34 ^b^	0.593	0.006
Tryptophan	1.70 ± 0.23 ^a^	1.03 ± 0.43 ^b^	1.05 ± 0.39 ^b^	0.108	0.008
Valine	13.74 ± 3.03 ^a^	8.25 ± 2.14 ^b^	8.37 ± 1.41 ^b^	0.802	0.001
γ-amino-*n*-butyric acid	0.008 ± 0.004	0.008 ± 0.007	0.012 ± 0.008	0.002	0.609
Glycine	43.57 ± 4.07	35.76 ± 5.37	42.45 ± 7.14	1.508	0.064
Serine	24.45 ± 2.05 ^a^	17.89 ± 3.03 ^b^	20.72 ± 6.10 ^ab^	1.119	0.044
Taurine	65.17 ± 9.73 ^a^	48.97 ± 2.13 ^b^	51.17 ± 7.73 ^b^	2.371	0.003
Tyrosine	9.84 ± 0.69 ^a^	6.60 ± 1.40 ^b^	6.65 ± 1.23 ^b^	0.4.47	0.000
Asparagine	14.08 ± 1.68 ^a^	8.93 ± 1.45 ^b^	10.21 ± 2.98 ^b^	0.712	0.002
Aspartic acid	9.73 ± 2.75	8.09 ± 0.75	9.13 ± 1.74	0.457	0.354
Citrulline	1.98 ± 0.39	2.04 ± 0.53	2.22 ± 0.56	0.113	0.711
Glutamic acid	36.74 ± 1.82 ^a^	30.55 ± 3.86 ^b^	33.28 ± 5.22 ^ab^	1.058	0.046
Glutamine	331.46 ± 51.9 ^a^	206.98 ± 41.1 ^b^	226.69 ± 40.2 ^b^	16.542	0.000
Ornithine	4.16 ± 0.53 ^a^	2.80 ± 0.58 ^b^	3.12 ± 0.90 ^b^	0.208	0.010
Cystine	1.11 ± 0.16	0.91 ± 0.32	0.84 ± 0.41	0.075	0.322
α-amino-*n*-butyric acid	379.51 ± 120.9	281.96 ± 63.6	343.98 ± 66.4	21.738	0.184
Alanine	32.66 ± 3.01 ^a^	26.33 ± 4.10 ^b^	29.81 ± 4.48 ^ab^	1.070	0.043
hydroxy-l-proline	14.58 ± 0.97	15.55 ± 2.03	14.37 ± 1.26	0.352	0.362
1-methyl-l-histidine	0.11 ± 0.05	0.10 ± 0.03	0.07 ± 0.02	0.009	0.188
3-methyl-l-histidine	0.04 ± 0.01 ^a^	0.02 ± 0.01 ^b^	0.03 ± 0.02 ^ab^	0.004	0.028
Proline	23.76 ± 1.55 ^a^	18.17 ± 3.36 ^b^	18.44 ± 4.38 ^b^	0.962	0.017

^a,b^ Means in the same row with different superscripts differ (*p* < 0.05). ^1^ Control = basal diet; ^2^ DON = basal diet + 6 mg/kg deoxynivalenol; and ^3^ DON + ARG = basal diet + 6 mg/kg deoxynivalenol + 1% l-arginine. Results are expressed as the means ± SEM for six animals.

### 2.4. Jejunal Morphology Changes

**Figure 1:**
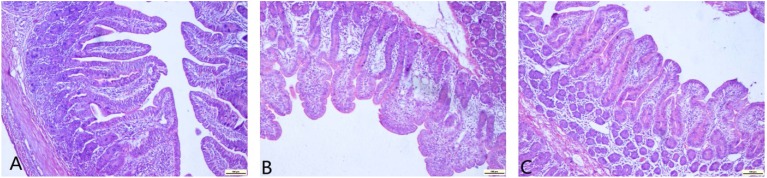
Effects of DON exposure on histopathology in the jejunum of weanling pigs: (**A**) normal histological structure in pigs fed the control diet; (**B**) severely impaired enterocytes in pigs fed the 6 mg/kg DON diet; (**C**) mild impairment of enterocytes in pigs fed the 6 mg/kg DON + 1% arginine diet. Original magnification: 400× (*n* = 6). All the scale bars in figures A to C represent 100 µM.

Pathological changes (Table 5) and deformation of enterocytes (Figure 1) in the mucosa were observed after the DON-infected diet was fed to pigs. Of note, only slight changes were found in the DON + ARG group. The villus height in the DON group was significantly lower than the other groups. The decrease in the DON + ARG group was mild. No difference was found between the DON + ARG and control groups (*p* < 0.05). No significant changes were found in crypt depth among the three groups of pigs. The ratio of villus height to crypt depth in the DON group was the lowest. This ratio in the DON + ARG group was slightly decreased compared to the control group (*p* > 0.05).

**Table 5:** Villus height and crypt depth in the pig jejunum after deoxynivalenol (DON) exposure (*n* = 6).

Items	Control ^1^	DON ^2^	DON + ARG ^3^	SEM	*p*-Value
villus height (μM)	250.3 ± 23.2 ^a^	198.7 ± 31.4 ^b^	228.5 ± 26.8 ^ab^	14.955	0.0173
crypt depth (μM)	102.4 ± 11.7	92.7 ± 14.2	97.6 ± 10.3	2.800	0.4080
villus height/crypt depth	2.46 ± 0.21 ^a^	2.13 ± 0.19 ^b^	2.35 ± 0.15 ^ab^	0.096	0.0224

^a,b^ Means in the same row with different superscripts differ (*p* < 0.05). ^1^ Control = basal diet; ^2^ DON = basal diet + 6 mg/kg deoxynivalenol; and ^3^ DON + ARG = basal diet + 6 mg/kg deoxynivalenol + 1% l-arginine. Results are expressed as the means ± SEM for six animals.

### 2.5. Expression of Nutrient Transporters

The mRNA levels for intestinal nutrient transporters are shown in Table 6. Significant differences were observed in the expression of sodium-glucose transporter-1 (SGLT-1) among three groups (*p* < 0.01), with the lowest values in the DON group. Compared to the control, other groups had lower mRNA levels for glucose transporter type-2 (GLUT-2) and y^+^l-type amino acid transporter-1 (y^+^LAT-1), but only slight differences in y^+^LAT-1 mRNA expression were detected between the control and DON + ARG groups. However, there were no differences in B^0,+^AT and peptide transporter-1 (PepT-1) expression noted among the three groups of pigs (*p* > 0.05).

## 3. Discussion

DON is a common contaminant of cereal crops, like wheat, barley, corn and oats, and of high importance in the food and feed industry and, increasingly, a food safety issue problem worldwide. Some reports suggested that ingestion of DON may induce feed refusal, decreased animal productivity, organ damage, increased disease incidence and malabsorption of nutrients [1,2,3,4,5,6,7,15,16,17]. The integrity of jejunum mucosal morphology and structure is a prerequisite of biological functions [16,17]. The villus height and crypt depth represent the metabolic and mature status of intestinal epithelial cells [18]. We found that DON damaged the integrity of the small intestine, but had no effect on the crypt depth. These results indicated that DON affects intestinal health via other ways, rather than harassing the development of intestinal cells. Meanwhile, no notable differences in the villus height or the value of villus height/crypt depth were found between the DON + ARG and control groups. According to previous studies, Arg supplementation in diet increases the intestinal mucosal thickness and the number of the small intestinal villi [19,20]; Arg stimulates the hypothalamus to release growth hormone, so as to reduce intestinal mucosa atrophy, accelerate injury recovery and maintain the structure and function of the intestinal mucosa [21]. We, therefore, conclude that Arg ameliorates the changes of the intestinal mucosal morphology and structure caused by DON.

**Table 6:** mRNA levels for nutrient transporters after deoxynivalenol (DON) challenge (*n* = 6).

Items	Control ^1^	DON ^2^	DON + ARG ^3^	SEM	*p*-Value
SGLT-1 ^4^	1.38 ± 0.08 ^a^	0.68 ± 0.05 ^c^	0.81 ± 0.09 ^b^	0.215	<0.0001
GLUT-2 ^5^	1.00 ± 0.08 ^a^	0.66 ± 0.13 ^b^	0.78 ± 0.15 ^b^	0.100	0.0009
y^+^LAT-1 ^6^	1.00 ± 0.12 ^a^	0.75 ± 0.09 ^b^	0.97 ± 0.10 ^a^	0.079	0.0015
ASCT-2 ^7^	1.07 ± 0.19 ^a^	0.98 ± 0.07 ^b^	1.02 ± 0.13 ^b^	0.026	0.5450
B^0,+^AT ^8^	1.26 ± 0.13	1.14 ± 0.080	1.12 ± 0.180	0.044	0.1909
PepT-1 ^9^	1.16 ± 0.15	1.07 ± 0.12	1.21 ± 0.09	0.041	0.1681

^a,b,c^ Means in the same row with different superscripts differ (*p* < 0.05). ^1^ Control = basal diet; ^2^ DON = basal diet + 6 mg/kg deoxynivalenol; and ^3^ DON + ARG = basal diet + 6 mg/kg deoxynivalenol + 1% l-arginine; ^4^ sodium-glucose transporter-1; ^5^ glucose transporter type-2; ^6^ y^+^l-type amino acid transporter-1; ^7^ Na^+^-dependent neutral amino acid transporter-2; ^8^ B^0,+^ amino acid transporter; ^9^ dipeptide transporter-1. Results are expressed as the means ± SEM for six animals.

Amino acids provide the basic raw material for protein synthesis. In the present study, DON treatment decreases the levels of some amino acids, including histidine and isoleucine. The possible explanation for this is the damage of DON to the intestinal tract. Indeed, previous studies have found that mycotoxin prevented the absorption of amino acids [22]. Similarly, T-2 toxin has been reported to reduce the absorption of amino acids, resulting in lower plasma concentrations [23]. In line with these well-designed investigations, we observed lower concentration of isoleucine and valine in the serum, jejunum and ileum of DON-challenged pigs. Isoleucine and valine function to repair wounds, regulate glutamine and arginine synthesis and provide energy to body tissues [24,25]. Previous evidence has indicated that Arg is an important factor for maintaining the mucosal integrity of the intestine and normal physiology of the gastrointestinal tract via improving the development of enterocytes [26]. It is not surprising that Arg enhances the absorption of amino acids, because of, at least partially, the repair function of Arg on the intestine. Intriguingly, the serum concentration of tryptophan was reduced significantly after DON treatment. Because tryptophan is not synthesized by animal cells, it would be of interest to explore the effect of dietary supplementation with tryptophan on pigs treated with DON.

The absorption of amino acids relies on the capability and amounts of relative transport carriers. The abnormal expression of transporters led to severe absorption defects and metabolic diseases [27]. In our previously study, we had found that DON-infected feed reduced the mRNA expressions of excitatory amino acid carrier 1 and cationic amino acid transporter in 60 d-old pigs [28]. In the present work, the mRNA levels for ASCT-2, y^+^LAT-1, B^0,+^AT and PepT-1 were determined to investigate the effect of DON-infected feed on intestinal expression of additional transporters. Our results showed that DON significantly reduced the expression of y^+^LAT-1, a transporter that transfers isoleucine, leucine, valine, phenylalanine and other macromolecular ranched chain and aromatic neutral amino acids in the jejunum mucosa [29]. This may be a possible explanation for the decreased concentrations of amino acids in serum and gut after exposure to DON. Since the expression and activity of y^+^LAT-1 are affected by the nutrient supply [30], a reduction in tissue protein synthesis may contribute to decreased expression of y^+^LAT-1 after DON exposure. Collectively, our results demonstrated that Arg affected y^+^LAT-1 expression under the stress state. However, there is no significant difference in mRNA levels for ASCT-2, B^0,+^AT and PepT-1 in the small intestine after DON exposure and Arg supplementation. The signaling pathway and mechanisms remain to be elucidated.

The absorption of glucose relies on two types of transporters in the small intestine: (i) Na^+^-coupled glucose transporters (SGLT-1) on the apical membrane; and (ii) facilitated glucose transporters (e.g., GLUT-2) that regulate the basolateral exit of glucose. The efficiency of glucose transport is affected by the activity and abundance of SGLT-1 [31]. At a higher glucose concentration, GLUT-2 may also participate in apical glucose absorption [32,33]. In the present work, there is no difference in the mRNA level of GLUT-2, whereas the mRNA level of SGLT-1 was decreased after DON exposure. GLUT-2 mediates the balance of glucose concentration in the intestine. At lower glucose concentrations, GLUT-2 transfers glucose from the blood to intestinal epithelial cells [34]. The reason why no change in mRNA levels for GLUT-2 occurred in the three groups of pigs is unknown, and this result may be explained by no differences in the concentrations of glucose or amino acids in the lumen of the small intestine. When Shepherd *et al.* analyzed the stress response of rats to noise and vibration, they observed an opposite result to our finding [35]. This difference may be due to the different stressors and distinct reaction mechanisms. The former study showed that Arg improved the absorptive capacity of glucose in the small intestine in the stress state [36,37]. In our study, SGLT-1 mRNA expression is significantly higher in the Arg group than the DON group, suggesting that Arg supplementation was able to alleviate the harm of DON on intestinal absorptive capacity. This finding supports our previous results that Arg protects intestinal health via sustaining the integrity of intestinal mucosa and villus [38,39]. Arg is truly a functional AA in animal nutrition and health [40].

## 4. Experimental Section

### 4.1. Preparation of Moldy Corn

*Fusarium graminearum* strain R6576 was supplied by Huazhong Agricultural University in China (Wuhan, Hubei Province, China) [28]. It was firstly cultivated on potato dextrose agar (PDA) at 28 °C for 7 days. The hyphae of the fungi were obtained and then inoculated in carboxymethyl cellulose (CMC) liquid medium for shaking cultivation (28 °C, 200 rpm/min, 5 days). This medium was extracted with acetonitrile. A blood-counting chamber was used to determine the number of conidia and set its concentration to 5 × 10^5^/mL. Finally, corn was spread on the indoor-ground, inoculated by CMC liquid culture (25 kg/L) for 7 days and then stored at −20 °C. The contents of mycotoxins in mold-contaminated feed were detected by liquid chromatography (Beijing Taileqi, Beijing, China) (Table 1).

### 4.2. Animals and Management

The study was conducted according to the guidelines of the Declaration of Helsinki. All procedures were approved by the animal welfare committee of the Institute of Subtropical Agriculture, Chinese Academy of Sciences (Changsha, Hunan Province, China). A total of eighteen, 28-day-old healthy weanling pigs (landrace × large × white) were randomly assigned into the control group (fed with uncontaminated basal diet), DON group (challenged by 6 mg/kg DON in diet) or DON + ARG group (6 mg/kg DON + 1% Arg group), respectively. There were 6 pigs per group (three male; three female). Piglets in the DON + ARG group were fed the basal diet supplemented with 1% Arg, whereas pigs in the control and DON groups were fed diets supplemented with 2.05% l-alanine as the isonitrogenous control. At the beginning of the experiment, all piglets were fed commercial diets with a concentration of DON less than 0.1 mg/kg. The composition of the basal feed is listed in Table 7. After 21 days of supplementation, the piglets in the DON and DON + ARG groups were challenged by feeding the 6 mg/kg DON-contaminated diet every day for a week. At the end of the 28-day experiment, all piglets were electrically stunned and slaughtered for analysis. Body weight and feed consumption were recorded.

**Table 7:** Composition and nutrient levels of diets.

Ingredients	Contents (%)	Nutrient Levels	Contents
Extrusion corn	60	Digestive energy, MJ/kg	14.48
Acidifier	0.24	Crude protein, %	20.90
Additive premix ^1^	0.85	Lysine·HCl, %	1.48
Glucose	3.2	Methionine, %	0.42
Fish meal	2	Threonine, %	0.90
Soybean meal	20	Calcium, %	0.80
CaHPO4	1.2	Available phosphorus, %	0.45
Limestone	1.19		
Soybean oil	2		
Lysine·HCl	0.28		
Threonine	0.04		

^1^ Premix provided the following per kilogram of the diet: vitamin A 2,000 IU; vitamin D_3_ 200 IU; vitamin E 12I U; vitamin K 0.5 mg; vitamin B_12_ 0.016 mg; vitamin B_2_ 3 mg; vitamin B_3_ 12.5 mg; folic acid 0.3 mg; vitamin B_5_ 10 mg; choline chloride 0.5 mg; vitamin B_1_ 1 mg; vitamin B_6_ 1.6 mg; vitamin B_7_ 0.05 mg; Cu 5 mg; Fe 80 mg; Mn 3 mg; Zn 85 mg; I 0.1 mg; Se 0.3 mg.

### 4.3. Sample Collection

After 28 days of dietary exposure to DON, 5 mL of blood were collected aseptically in tubes from a jugular vein 2 h after feeding, centrifuged at 3,000× *g* for 10 min at 4 °C to obtain serum samples and stored at –80 °C for further analysis. The small intestine was rinsed thoroughly with ice-cold physiological saline solution (PBS), and the jejunum and ileum were dissected.

### 4.4. Determination of Free Amino Acids Profile in Serum, Ileum and Jejunum

Free amino acids in serum were determined as previously described [24]. Briefly, a 500-μL sample was hydrolyzed in 10 mL 6 mol/L HCl at 110 °C for 24 h. The solution was then adjusted to the volume of 50 mL, and then, 1.0 mL of the settled solution was filtered through a 0.45-μM membrane for free amino acids analysis using an ion-exchange AA analyzer (Hitachi L-8800 Auto-Analyzer, Tokyo, Japan).

To measure the free amino acid profiles in ileum and jejunum, approximately 0.1 g freeze-dried ileum and jejunum were ground and added to 10 mL of 6 mol/L HCl for hydrolyzing at 110 °C for 24 h. The solution was then transferred to new Eppendorf tubes. After a 10-fold dilution, the samples were filtered through a 0.45-μM membrane for free amino acids analysis using an ion-exchange AA analyzer (Hitachi L-8800 Auto-Analyzer, Tokyo, Japan), as previously described [24].

### 4.5. Determination of Jejunal Morphology

After embedding in paraffin, the jejunum samples were sectioned into 2–4 cm slides parallel to the villi axis and stained by hematoxylin and eosin (H & E) using standard procedures [28]. After dehydration, embedding, sectioning and staining, villous height and crypt depth were measured with computer-assisted microscopy (Micrometrics TM; Nikon ECLIPSE E200, Tokyo, Japan).

### 4.6. RNA Extraction and cDNA Synthesis

Liquid nitrogen was used to pulverize the jejunum intestine sample. Total RNA was isolated from 100 mg of the homogenate using TRIzol reagent (Invitrogen, Carlsbad, CA, USA) and treated with DNase I (Invitrogen), according to the manufacturer’s instructions. The quality of RNA was checked by 1% agar gel electrophoresis, under staining with 10 mg/mL ethidium bromide. The OD_260_:OD_280_ ratio of RNA was between 1.8 and 2.0. First-strand cDNA was synthesized with Oligo (dT) 20 and Superscript II reverse-transcriptase (Invitrogen).

### 4.7. Quantification of mRNA by Real-Time RT-PCR Analysis

Primers were designed with Primer 5.0 based on the cDNA sequence of the pig to produce an amplification product (Table 8). β-actin was used as a housekeeping gene to normalize target gene transcript levels. Real-time PCR analysis was performed as described previously [28]. In brief, 2 µL of cDNA template were added to a total volume of 25 µL containing 12.5 µL SYBR Green mix and 1 µmol/L each of forward and reverse primers. We used the following protocol: (i) pre-denaturation (10 s at 95 °C); (ii) amplification and quantification, 40 cycles (5 s at 95 °C, 20 s at 60 °C); and (iii) melting curve (60–99 °C with a heating rate of 0.1 °C·S^−1^ and fluorescence measurement). The relative levels of genes were expressed as a ratio of mRNA as R = 2^−(∆∆*C*t)^. The efficiency of real-time PCR was determined by the amplification of a dilution series of cDNA according to the equation 10^(−1/slope)^, and the results for target mRNA were consistent with those for β-actin. Negative controls were created by replacing cDNA with water.

### 4.8. Statistical Analysis

All data, expressed as the mean ± standard error of the mean (SEM), were subjected to ANOVA analysis using the SPSS 13.0 software (SPSS, Chicago, IL, USA) [20]. The differences among group means were compared using the Duncan multiple comparison test. Probability values < 0.05 were taken to indicate statistical significance.

**Table 8:** Primers used for RT-PCR.

Target Gene	Primer Sequence (5' to 3')	Accession No.	Size
B^0,+^AT-F1 ^1^	GCGAGTACCCGTACCTGATG	NM_001110171.1	173
B^0,+^AT-R1	TTTCACGACGACTTGAGGGG		
SGLT1-F1 ^2^	TCATCATCGTCCTGGTCGTCTC	M34044.1	144
SGLT1-R1	CTTCTGGGGCTTCTTGAATGTC		
GLUT2-F1 ^3^	ATTGTCACAGGCATTCTTGTTAGTCA	NM_001097417	273
GLUT2-R1	TTCACTTGATGCTTCTTCCCTTTC		
y^+^LAT1-F1 ^4^	TTCTCTTACTCGGGCTGGGA	EU047705.1	400
y^+^LAT1-R1	GCGCCATGAGACCATTGAAC		
GAPDH-F1 ^5^	AAGGAGTAAGAGCCCCTGGA	DQ845173	140
GAPDH-R1	TCTGGGATGGAAACTGGAA		
ASCT2-F1 ^6^	CTGGTCTCCTGGATCATGTGG	DQ231578.1	172
ASCT2-R1	CAGGAAGCGGTAGGGGTTTT		
PepT1-R1 ^7^	CAGACTTCGACCACAACGGA	NM_214347.1	99
PepT1-F1	TTATCCCGCCAGTACCCAGA		

^1^ B^0,+^ amino acid transporter; ^2^ sodium-glucose transporter-1; ^3^ glucose transporter type-2; ^4^ y^+^ l-type amino acid transporter-1; ^5^ glyceraldehyde-3-phosphate dehydrogenase; ^6^ Na^+^-dependent neutral amino acid transporter-2; ^7^ dipeptide transporter-1.

## 5. Conclusions

In conclusion, weanling pigs fed a diet containing 6 mg/kg DON demonstrated an adverse effect on intestinal morphology, permeability and absorption function. Additionally, dietary supplementation with Arg alleviates the impairment in the intestinal tract brought about by DON challenge. Thus, Arg exerts a protective role against DON in pigs. These results may also have important nutritional implications for humans and other mammals.
